# Scientists and scientific organizations need to play a greater role in science diplomacy

**DOI:** 10.1371/journal.pbio.3001848

**Published:** 2022-11-01

**Authors:** Peter D. Gluckman

**Affiliations:** 1 International Science Council, Paris, France; 2 Koi Tū: The Centre for Informed Futures, University of Auckland, Auckland, New Zealand

## Abstract

Science diplomacy is critical for addressing global challenges such as climate change and pandemics. In this Perspective, Peter Gluckman argues that scientists and scientific organizations have important informal diplomatic roles to play beyond the formal processes of science diplomacy.

## Teaser

Science diplomacy—the use of science to advance diplomatic goals—is critical for addressing challenges to the global commons such as climate change and pandemics. Beyond the formal processes of science diplomacy, scientists and scientific organizations can play important informal diplomatic roles.

We live in perilous times, with informed commentary being dominated by pandemics, climate change, environmental degradation, collapse of the post–World War II rules-based system, growing nationalism, declining social cohesion, and disinformation. At the same time, progress on the Sustainable Development Goals has been inadequate [1]. The broader consequences of this confluence of factors for citizens in every part of the world have manifested as increased inequalities, loss of opportunity, greater mental health concerns, and greater fragility for many lives.

Science and technology have had causal roles in these evolving challenges, but will be critical to finding solutions. The sciences and their technological offspring contributed to the marked extension in lifespan that all societies have seen in the past 100 years. But the very technologies that have been developed also gave rise to greenhouse gas emissions and advanced weapons. As scientists argue for the use of new technologies and the better application of much existing knowledge to address the many challenges ahead, we must ensure societies are appropriately engaged.

Many of these issues transcend national borders or are common across countries. Science diplomacy, the use of science to advance diplomatic goals, is an important strategy in effecting better engagement. Such diplomacy can be parsed by whether those goals are to advance a nation’s direct interests (e.g., in security, trade, or projecting soft power) or whether they commit to addressing the challenges to the global commons [2]. The tragedy of growing nationalism is that it can override the latter as a priority, as has been illustrated in the responses to climate change, to issues surrounding the Arctic, and the management of ocean resources. Each of these—and, indeed, aspects of the response to Coronavirus Disease 2019 (COVID-19) [3]—shows how we confront a fragile future when transnational cooperation is weak.

Yet, even in difficult times, diplomatic advances can be made. The first cold war was not devoid of major diplomatic advances, many of which originated with science [4]. The International Geophysical Year (1957), organized by the International Council of Scientific Unions (ICSU; the International Science Council’s (ISC) predecessor organization), was a multinational effort that led to many discoveries such as the mid-oceanic ridges confirming the theory of continental drift. The focus on scientific cooperation in the Antarctic led to the Antarctic Treaty (1959), to which all major countries are now signatories, and which restricts activities in the Antarctic to peaceful (scientific) purposes; this remains coordinated through the ISC affiliate body, the Scientific Committee on Antarctic Research. Similarly, the Villach conference (1985), convened by ICSU with United Nations (UN) Environment Programme (UNEP) and the World Meteorological Organization, brought leading climate scientists together and led to the formation of the IPCC (1988). The landmark Montreal Protocol on Substances that Deplete the Ozone Layer (1987) was possible because science and technology had highlighted the problem and identified solutions. The formation of the International Institute of Applied Systems Analysis (1962) was an initiative of the United States President and the Soviet Premier to use science to reduce tensions between the 2 superpowers. These, and many other examples including the collaborative efforts to eradicate smallpox and polio, highlight how science was and can continue to be an important tool of both formal (“Track 1”) and informal (“Track 2”) global diplomacy.

Unfortunately, the current multilateral system is not functioning well. Yet, so many issues require the global community, represented by the UN, to use science and knowledge more effectively. Indeed, this was identified by the UN Secretary-General in his 2021 report to the General Assembly [5]. The ISC, whose members comprise national scientific academics and multinational scientific unions and associations, is the primary nongovernmental organization representing science in the multilateral arena; ISC has been interacting with the offices of the Secretary-General and the President of the General Assembly to discuss what kind of mechanisms might assist. This would be greatly facilitated if more foreign ministries gave stronger emphasis to science advice within their ministries [2], given that the UN is an instrument of its member states. Individual UN agencies are variable in how they approach the use of science; some have their own science advisors and advisory groups, but their structure and links to the global science community are variable. The UNEP is to be congratulated on its recent analysis of these issues [6]; it is working with the ISC to strengthen its own capacities at the science–policy interface and to engage a diverse plurality of natural and social scientists in their work.

Many foreign ministries view science narrowly, looking for economic and technological advantage or simply cultural promotion. However, a small but growing number including the United Kingdom, USA, and Japan have appointed science advisors as formal positions within their foreign ministries. This has broadened their capacities to tackle the range of issues requiring scientific input at the diplomatic table. Initially, this was largely in the security space, but foreign ministries have increasingly needed to focus on issues of environment, climate change, biodiversity loss, and technological developments. Other issues, including that of the ungoverned spaces, such as the regulation of oceans and inner and outer space, continue to need more effective scientific input into policy making. A growing focus must be on the raft of emergent life science and digital associated technologies that might affect national interests, have no jurisdictional boundaries (e.g., genetically manipulated organisms), and could have geopolitical consequences. The scientific community needs to be more active here both within and between countries. The development of the Geneva Science and Diplomacy Anticipator (GESDA) as a forum to project how evolving technologies will impact diplomacy illustrates this. Its radar presents a consensual scientific view on how technologies may evolve to allow an analysis of their broader societal and diplomatic consequences [7].

There is often a gulf between how ministries of science and foreign affairs view international science and its potential. Yet, all scientific cooperation has diplomatic spillover. The reaction of Atlantic states to the Ukrainian conflict by cutting off scientific ties involving funding illustrates this [8]. When the Ukraine crisis broke out, the ISC was confronted with the issue of whether to suspend the Russian member. After ethical consultation, a decision was made not to do so [9]. The logic was simple: No matter how much we deplore conflict, at some point, relationships must again be reestablished for the global good as was illustrated in the last cold war [2]. As a nongovernmental organization, we can achieve outcomes, which, in the current context, cannot be achieved by more formal processes. This decision was later supported in conversations with science diplomats.

In the current geopolitical context, informal or “Track Two” diplomatic activities including those undertaken by a variety of institutions will be essential to progress on issues of the global commons (Box 1). The ISC is reorienting its priorities to focus on its obligations in science diplomacy. This includes building more effective relations with the UN, its agencies, and other multilateral organizations, as well as building capacities in its own members to engage in science diplomacy. In many ways, the skills needed at the science-diplomacy interface are similar to those needed in brokerage between science and policy [10,11]. Science, scientists, and scientific organizations all have important roles to play as we face a challenging future.

**Box 1. “Track Two” diplomatic activities—building capacity and capability in science diplomacy**

Depending on the context, individuals, institutions, scientific organizations including academies and scientific unions can all contribute to science diplomacy. Any form of scientific collaboration can have diplomatic spillovers, but in order to engage beyond capricious impacts, it is important to understand the science–diplomatic interface just as with other aspects of the science–policy interface. Universities can have a critical role in bridging the science, public policy, and diplomatic communities as well as offering research and training opportunities in science diplomacy.

Organizations such as the International Network for Governmental Science Advice (INGSA), GESDA, American Association for Advancement of Science (AAAS) in conjunction with The World Academy of Sciences (TWAS), and an increasing number of universities provide short courses or summer schools that are aimed at early career scientists. Many diplomatic academies have extended their training beyond diplomats and promoted science diplomacy with the science community (e.g., Diplomatic Academy of Vienna). Some countries have followed the example of the National Academies of Sciences, Engineering, and Medicine’s Jefferson Science Fellowships and the AAAS’s Science and Technology Policy Fellowships, which allow scientists to work within foreign ministries. Such experiential engagement, perhaps through an internship, gives a greater understanding of the 2 cultures.

Science diplomacy has its own online journal for researchers (Science Diplomacy). The European Commission has supported major collaborative research efforts to develop and understand science diplomacy. Moreover, the same tools of science diplomacy need not refer only to nation-to-nation interactions, but also to cities and regions (e.g., the Barcelona SciTech DiploHub).

## Abbreviations

AAAS: American Association for Advancement of Science
COVID-19: Coronavirus Disease 2019
GESDA: Geneva Science and Diplomacy Anticipator
ICSU: International Council of Scientific Unions
INGSA: International Network for Governmental Science Advice
ISC: International Science Council
TWAS: The World Academy of Sciences
UNEP: United Nations Environment Programme
